# Identification and Characterization of a Novel Di-(2-ethylhexyl) Phthalate Hydrolase from a Marine Bacterial Strain *Mycolicibacterium phocaicum* RL-HY01

**DOI:** 10.3390/ijms26178141

**Published:** 2025-08-22

**Authors:** Lei Ren, Caiyu Kuang, Hongle Wang, John L. Zhou, Min Shi, Danting Xu, Hanqiao Hu, Yanyan Wang

**Affiliations:** 1 College of Coastal Agricultural Sciences, Guangdong Ocean University, Zhanjiang 524088, China; renlei@gdou.edu.cn (L.R.);; 2 Faculty of Science and Engineering, University of Nottingham Ningbo China, Ningbo 315100, China

**Keywords:** di-(2-ethylhexyl) phthalate, biodegradation, hydrolase, enzymatic properties, site-direct mutagenesis

## Abstract

Phthalic acid esters (PAEs), ubiquitously employed as a plasticizer, have been classified as priority environmental pollutants because of their persistence, bioaccumulation, and endocrine-disrupting properties. As a characterized PAE-degrading strain of marine origin, *Mycolicibacterium phocaicum* RL-HY01 utilizes di-(2-ethylhexyl) phthalate (DEHP) as its sole carbon and energy source. Genome sequencing and RT-qPCR analysis revealed a previously uncharacterized hydrolase gene (*dehpH*) in strain RL-HY01, which catalyzes ester bond cleavage in PAEs. Subsequently, recombinant expression of the cloned *dehpH* gene from strain RL-HY01 was established in *Escherichia coli* BL21(DE3). The purified recombinant DehpH exhibited optimal activity at 30 °C and pH 8.0. Its activity was enhanced by Co^2+^ and tolerant to most metal ions but strongly inhibited by EDTA, SDS, and PMSF. Organic solvents (Tween-80, Triton X-100, methanol, ethanol, isopropanol, acetone, acetonitrile, ethyl acetate, and *n*-hexane) showed minimal impact. Substrate specificity assay indicated that DehpH could efficiently degrade the short and long side-chain PAEs but failed to hydrolyze the cyclic side-chain PAE (DCHP). The kinetics parameters for the hydrolysis of DEHP were determined under the optimized conditions, and DehpH had a *V_max_* of 0.047 ± 0.002 μmol/L/min, *K_m_* of 462 ± 50 μmol/L, and *k_cat_* of 3.07 s^−1^. Computational prediction through structural modeling and docking identified the active site, with mutagenesis studies confirming Ser228, Asp324, and His354 as functionally indispensable residues forming the catalytic triad. The identification and characterization of DehpH provided novel insights into the mechanism of DEHP biodegradation and might promote the application of the target enzyme.

## 1. Introduction

Phthalic acid esters (PAEs) are a group of artificially synthesized chemical compounds that are mainly added into polymers to increase their flexibility and durability [1]. Due to their good plasticizing properties and low cost, they make up the highest global plasticizers market proportion, and it is estimated that over 10 million tons of PAEs have been consumed worldwide in 2020 (data from https://www.plasticisers.org/plasticisers and https://www.statista.com, accessed on 10 March 2025). However, PAEs could be easily released from the polymers since PAEs are not chemically bonded with matrices (known as external plasticizers), leading to their ubiquitous detection in various environments [2]. In addition, lots of works have demonstrated that PAEs could be accumulated in plants and resultantly enter the food chain [3,4]. Meanwhile, research evaluating PAE toxicity has identified their estrogenic and endocrine-disrupting effects, linking them to potential health impacts such as fertility problems, respiratory diseases, childhood obesity, abortion, and neuropsychological disorders [5,6,7]. As a result, six kinds of PAEs, including di-methyl phthalate (DMP), di-ethyl phthalate (DEP), di-n-butyl phthalate (DBP), di-n-octyl phthalate (DNOP), di-(2-ethylhexyl) phthalate (DEHP), and butyl benzyl phthalate (BBP), have been listed as priority pollutants by major environmental agencies, including the following: the United States Environmental Protection Agency [8], the European Union [9], and the China National Environmental Monitoring Center [10].

Considering the growing global consumption and potential health risks of PAEs, the fate of PAEs in different environmental matrices has raised great concern. Biodegradation serves as the principal pathway for PAE decomposition in nature and is considered the most promising strategy for eliminating these compounds from various environments [11]. During the last decades, a great number of PAE-degrading microbes have been isolated and characterized from aquatic, terrestrial, and marine ecosystems [2]. Among these microbes, bacteria always served as vital and dominant PAE degraders which typically utilize PAEs as essential nutrients and energy for growth. Both gram-positive and gram-negative bacteria, capable of degrading PAEs, have been widely reported, and the genera *Pseudomonas* [12], *Gordonia* [10], *Rhodococcus* [13], *Mycobacterium* [14], and *Sphingomonas* [15] were found to be the prevalent taxa. Further, the metabolic intermediates of PAEs have been systematically identified via mass-spectrometric analysis, and the metabolic pathways of PAEs were deduced thereafter [2,16]. PAE metabolism generally involves two key phases: upstream (transformation to phthalic acid) and downstream (phthalic acid utilization) [2]. Microbial degradation of aromatic compounds has been systematically reviewed by Fuchs et al., and known studies have demonstrated that phthalic acid might be utilized via catechol, protocatechuate, and gentisate [17]. Owing to their fundamental importance in decomposition, the upstream processes are a major focus for environmental and life science researchers. Typically, esterases catalyze the key upstream reactions involving PAE ester bond hydrolysis [2]. Therefore, discovering novel esterases capable of cleaving these bonds and elucidating their mechanisms are crucial for understanding PAE fate in diverse environments and devising sustainable remediation strategies for contaminated sites.

From mangrove sediments, we previously isolated *Mycolicibacterium phocaicum* RL-HY01, a novel bacterium capable of degrading PAEs [18]. Strain RL-HY01 utilizes DEHP, DBP, DEP, and DMP as its only carbon and energy inputs. The DEHP catabolic pathways were determined by UHPLC-MS/MS profiling of key metabolic intermediates. Specifically, the identification of metabolic intermediates suggested that strain RL-HY01 could transform DEHP into DEP via β-oxidation, while DEP was hydrolyzed into phthalic acid (PA) by de-esterification. Finally, PA was utilized for cell growth via ring cleavage. Although the enzymes involved in the transformation of mono-alkyl phthalates (MAPs) to PA and utilization of PA have been preliminarily identified, the enzyme that catalyzes the transformation of di-alkyl phthalates to MAPs is still unknown. In this study, a novel di-alkyl phthalates ester bond hydrolase gene, *dephH*, was cloned from strain RL-HY01 and identified in *Escherichia coli* (*E. coli*) BL21 (DE3) via heterologous expression. The recombinant DehpH was purified and biochemically characterized. Further, the three-dimensional (3D) model of DephH was constructed, and the amino acid residues potentially participating in the catalysis were predicted by molecular docking. Site-directed mutagenesis was employed for the functional confirmation of the active-site residues.

## 2. Results

### 2.1. Biodegradability of PAEs by Strain RL-HY01

**Figure 1:**
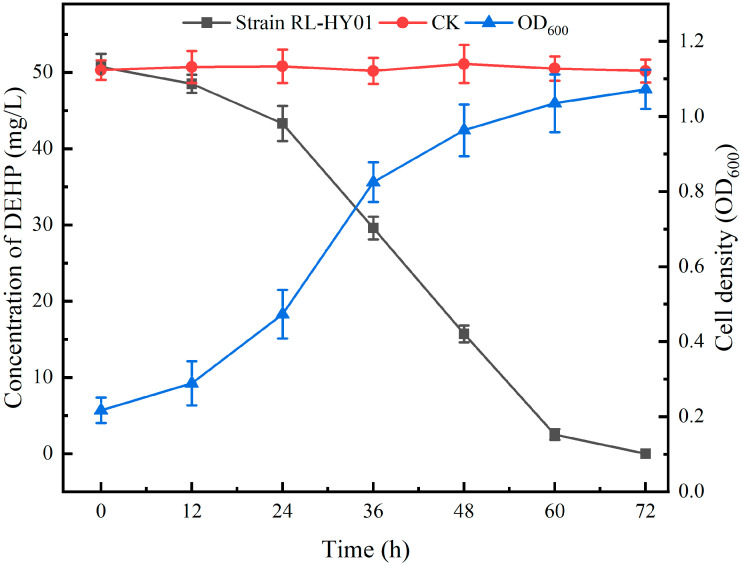
DEHP degradation curves and growth curve of strain RL-HY01. Error bars represent the standard errors for three replicates.

Strain RL-HY01 could utilize DEHP as the sole carbon source for growth. The degradation of DEHP was measured by GC, while the cell density was determined with a UV-visible spectrophotometer (Section 4.3). As shown in Figure 1, 50 mg/L DEHP could be completely degraded by strain RL-HY01 in 72 h, with the cell density increasing from 0.217 to 1.072. In the control treatment (CK), the concentration of DEHP did not decrease over the time of incubation, and no degradation was observed.

### 2.2. Genome Annotation and Bioinformatics Analysis

**Figure 2:**
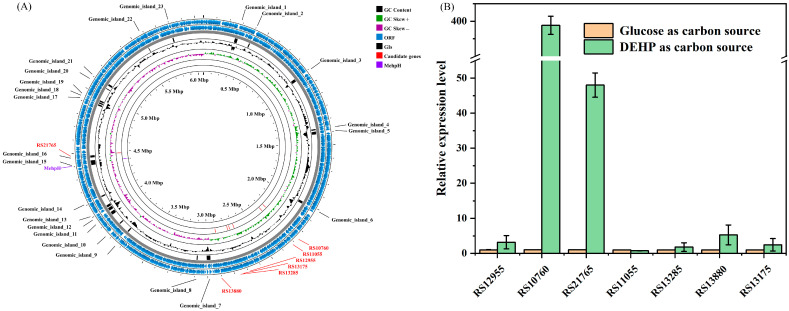
Circular map for the genome of strain RL-HY01 (**A**) and the expression level of the selected genes during the degradation of DEHP (**B**).

Following the genome extraction and quality control (Section 4.4), a total of 1,544,673,429 bp of clean data was generated with a ~254.7-fold coverage of the genome. After assembling and polishing, a circular chromosome of 6,064,759 bp (G + C content of 66.93 mol%) was obtained, and the general information of the genome sequence is shown in Table S3. Subsequently, 5874 genes were predicted, and 5681 of these genes were identified as protein coding sequences (96.7%). The annotation of predicted coding sequences (CDSs) against different databases was shown in Table S4. Further, in-depth analysis of genes potentially involved in the hydrolysis of ester bonds of PAEs was conducted. A known mono ethyl hexyl phthalate ester bond hydrolase (MehpH) coding sequence (locus tag: LHJ73_RS21370) was identified in the genome of strain RL-HY01. Nevertheless, none of the known DEHP ester bond hydrolase coding sequences was identified in the genome of strain RL-HY01. Therefore, to screen potential genes and genomic islands (GIs) involved in ester bond hydrolysis, we performed whole-genome BLAST alignment based on sequence similarity to known PAE hydrolases (Table S2). A total of seven candidate genes and 21 GIs were identified (Figure 2A).

### 2.3. RT-qPCR Analyses of Candidate Genes

To preliminarily verify the function of selected genes, the expression levels of these seven genes were determined during the degradation of DEHP (Section 4.5), and the results of RT-qPCR are shown in Figure 2B. Among these seven selected genes, only two of them (RS10760 and RS21765) were significantly upregulated at 24 h. When DEHP was supplied as the sole carbon source, the relative expression levels of RS10760 and RS21765 were 399 ± 3-fold and 48 ± 3-fold of the treatments that grew on glucose, respectively. However, for the other five selected genes, no significant differences in the relative expression level were observed between the treatments of glucose and DEHP. Consequently, RS10760 and RS21765 were selected as candidate genes for further functional confirmation.

### 2.4. Cloning and Heterologous Expression of Candidate Genes

**Figure 3:**
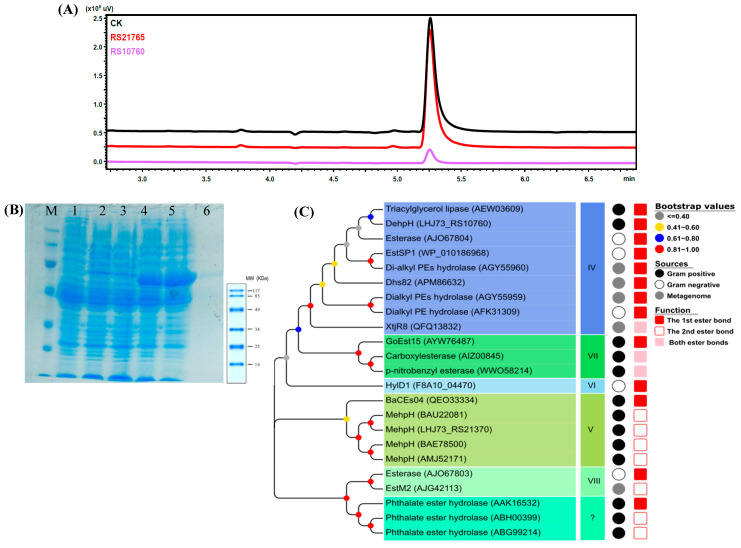
Characterization of the DEHP-degrading enzyme DehpH: activity, expression, purification, and phylogeny. GC analysis on the DEHP-degrading capability of the expressed candidate genes (**A**). SDS-PAGE analysis of DehpH expression and purification (**B**), Lane M: protein marker; lane 1: total protein of *E. coli* BL21(DE3); lane 2: total protein of *E. coli* BL21(DE3)-pET-32a without inducing; lane 3: total protein of *E. coli* BL21(DE3)-pET-32a induced by IPTG; lane 4: total protein of *E. coli* BL21(DE3)-pET-DehpH without inducing; lane 5: total protein of *E. coli* BL21(DE3)-pET-DehpH induced by IPTG; lane 6: purified recombinant DehpH. Phylogenetic analysis of DehpH and known enzymes capable of hydrolyzing the ester bonds of PAEs (**C**).

Genes of RS10760 and RS21765 were amplified from the genome of strain RL-HY01 and successfully cloned into pET28a(+) fused with an N-terminal Trx tag and a His6 tag for expression (Section 4.6). The constructed pET-RS10760 and pET-RS21765 were transformed into *E*. *coli* BL21 (DE3) for gene expression, separately. Subsequently, the expression of RS10760 and RS21765 was induced by IPTG, while the degradation of supplemented DEHP was determined by GC. The protein expression was preliminarily verified by SDS-PAGE prior to activity assays. Concurrently, the inducing of *E*. *coli* BL21 (DE3) containing pET28a(+) and the determination of DEHP degradation were conducted. The results were shown in Figure 3A. Approximately 96.31% of DEHP (50 mg/L) was degraded by pET-RS10760 in 8 h, while pET-RS21765 and pET28a(+) did not exhibit the capability to degrade DEHP. Consequently, RS10760 was identified as the gene coding for the DEHP ester bond hydrolase and was denominated as *dehpH*. To our knowledge, DehpH is the first reported DEHP ester bond hydrolase from *Mycolicibacterium* strains.

Concurrently, the *dehpH* gene was successfully inserted into pET28a(+) and transformed into *E*. *coli* BL21 (DE3) for gene expression. Subsequently, the DehpH gene was induced by IPTG, and the recombinant DehpH was purified using His60 Ni Magnetic Beads (TaKaRa, Beijing, China). The SDS-PAGE analysis revealed that a lane of protein with a molecular weight of ~46 kDa was obtained (Figure 3B). The recombinant DehpH most likely corresponded to the 6 kDa of His-Thrombin tag (N-terminal) and His tag (C-terminal) sequences fused to the expected 40 kDa of the *dehpH* gene product.

### 2.5. Biochemical Characterization of DehpH

**Figure 4:**
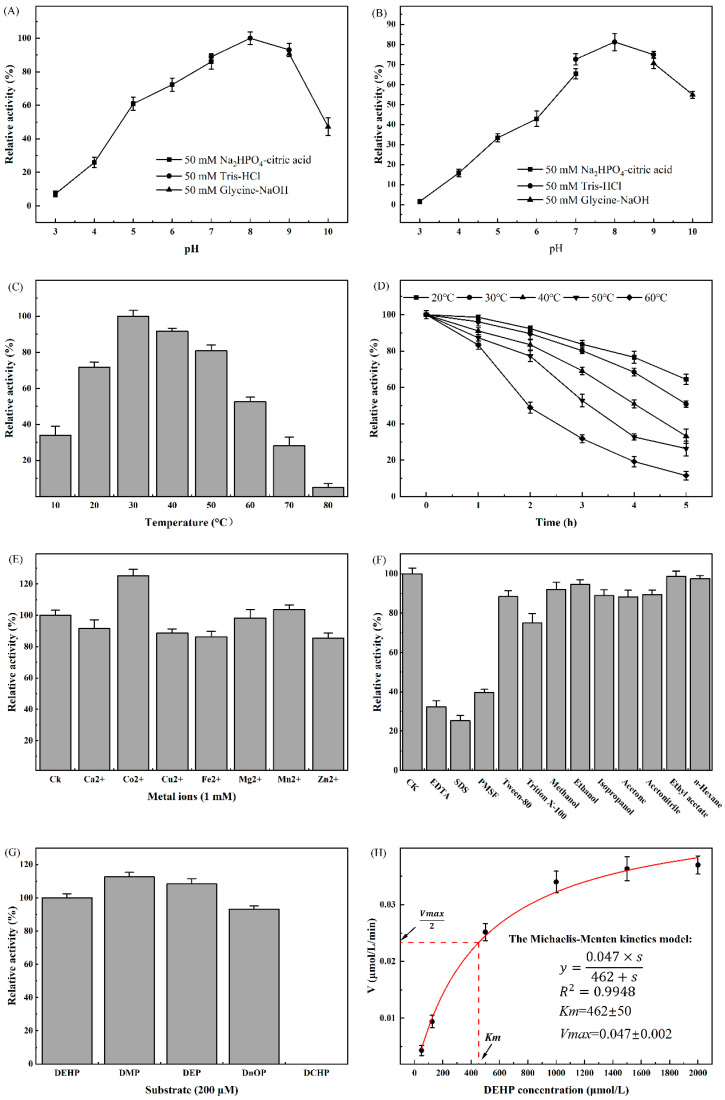
Biochemical characterization of DehpH. (**A**): effects of pH on enzyme activity; (**B**): effects of pH on enzyme stability; (**C**): effects of temperature on enzyme activity; (**D**): effects of temperature on enzyme stability; (**E**): effects of metal ions on enzyme activity; (**F**): effects of surfactants and chemicals on enzyme activity; (**G**): substrate specificity of DehpH; (**H**): kinetics parameters of DehpH.

The effects of different environmental factors on the activity of DehpH were determined, and the optimal conditions for DehpH were proposed thereafter (Section 4.8). The effects of pH on the activity of DehpH were shown in Figure 4A. The results indicated that the optimal pH was 8.0, and DehpH showed relatively high activity ranging from pH 6.0 to pH 9.0. After incubation at 4 °C under different pH for 1 h, the activity of DehpH decreased significantly under acid conditions (pH 3.0 to 6.0), and DehpH almost lost all of its activity under pH 3.0, while DehpH retained more than 70.0% residual activity under pH 7.0 to 9.0 (Figure 4B). The activity of DehpH increased in the temperature range of 10 °C to 30 °C and showed maximum activity at 30 °C (Figure 4C). Subsequently, the activity of DehpH decreased in the temperature range from 30 °C to 80 °C, and DehpH almost lost its activity under 80 °C. The thermostability experiment showed that the activity of DehpH decreased with the increasing of the incubation time, and the higher the temperature, the greater the loss of activity (Figure 4D). After 5 h incubation, approximately 64.0% of DehpH activity was retained under 20 °C, while only 11.4% of DehpH activity was there under 60 °C.

The effects of metal ions on the activity of DehpH were shown in Figure 4E. The results demonstrated that Co^2+^ could significantly promote the activity of DehpH, while Mg^2+^ and Mn^2+^ showed no significant influence on the activity of DehpH. On the contrary, Ca^2+^, Cu^2+^, Fe^2+^, and Zn^2+^ could decrease the activity of DehpH by 8.5% (Ca^2+^) to 15.4% (Zn^2+^). DehpH showed weak tolerance to most of the selected surfactants (Figure 4F). DehpH maintained 75.03% and 88.40% enzyme activities in the presence of 1% (*v*/*v*) Tween-80 and Triton X-100, respectively, but its activity dramatically declined in the presence of 1% (*v*/*v*) EDTA, SDS, and PMSF. However, the selected chemicals did not significantly affect the activity of DehpH, with the retained activities all above 88.0% (Figure 4F). The substrate specificity analysis (Figure 4G) suggested that DehpH can degrade the short and long side-chain PAEs but failed to degrade cyclic side-chain PAE (DCHP). Further, the kinetics parameters of DehpH were determined with the Michaelis–Menten kinetics model under the optimized conditions (Figure 4H). The values of *V_max_*, *K_m_*, and *k_cat_* were 0.047 ± 0.002 μmol/L/min, 462 ± 50 μmol/L, and 3.07 s^−1^, respectively.

### 2.6. Structural Modeling and Molecular Docking of DehpH

**Figure 5:**
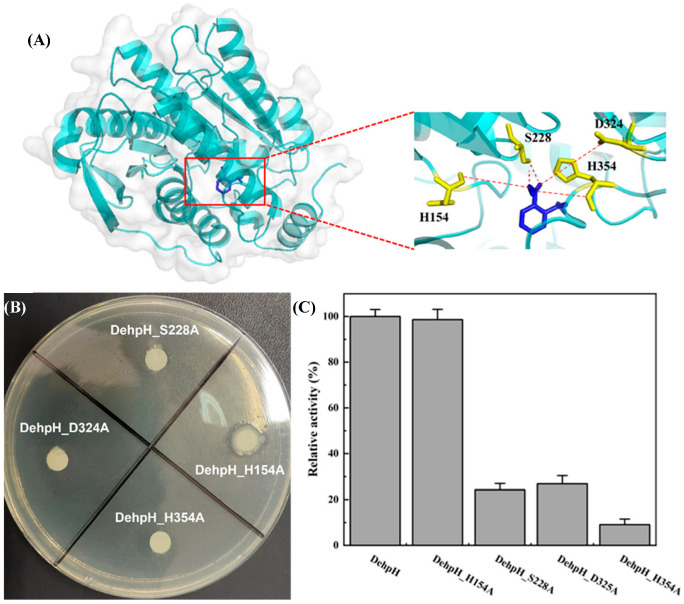
Identification of catalytic triad. Three-dimensional structure modeling and molecular docking of DehpH (**A**); site-directed mutagenesis of His154, Ser228, Asp324, and His354 (**B**); comparison of relative enzyme activity between DehpH and its mutants (**C**).

Prior to molecular docking, the predicted 3D models of DehpH were checked by SAVEs v6.0, and the one with the highest scores was selected for further study (Section 4.9). The selected 3D model was visualized by Pymol and shown in Figure 5A. Molecular docking of receptor DehpH and ligand DMP was conducted with Autodock, and the conformation with the lowest free energy was applied for the analysis of protein-ligand interactions. The results of molecular docking showed that DMP was located in an active pocket of DehpH, and several amino acid residues might form hydrogen bonds with the substrate to stabilize the substrate or initialize nucleophilic attack, including His154, Ser228, Asp324, and His354.

### 2.7. Site-Directed Mutagenesis of the Active-Site Residues

**Figure 6:**
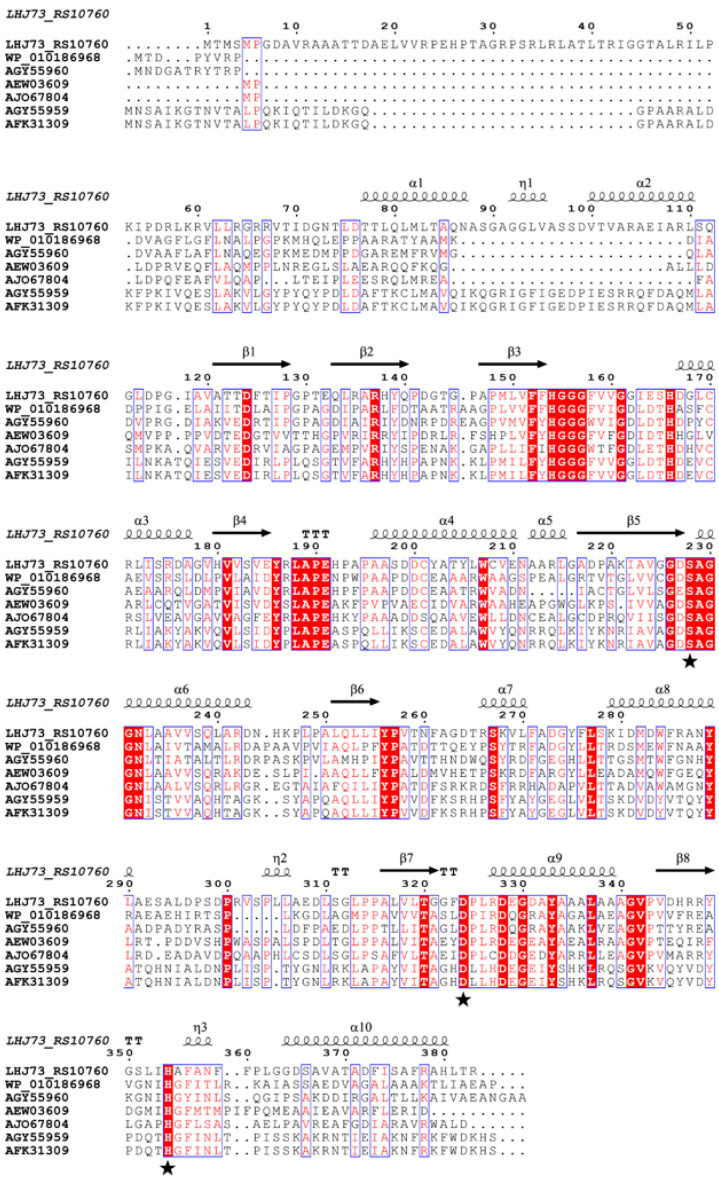
Multiple sequence alignment of DehpH with the esterases of family IV. The accession numbers are associated with the esterases of family IV shown in Figure 3C. α, β, and η represent the protein secondary structures α-helix, β-sheet, and η-helix, respectively, with asterisks denoting the three amino acid residues Ser228, Asp324, and His354.

According to the results of multiple sequence alignment (Figure 6) and molecular docking (Section 4.10), the potential amino acid residues of the catalytic triad were proposed as His154, Ser228, Asp324, and His354. Subsequently, site-directed mutagenesis of these amino acid residues was carried out by replacing them with alanine, respectively. As shown in Figure 5B, a clear hydrolysis halo appeared around the colony of *E*. *coli* BL21 (DE3)-DehpH_H154A, while *E*. *coli* BL21 (DE3)-DehpH_S228A, *E*. *coli* BL21 (DE3)-DehpH_D325A, and *E*. *coli* BL21 (DE3)-DehpH_H354A failed to hydrolyze DEHP in plates. Moreover, the recombinant DehpH and its mutants were purified, and their relative activities were measured. The results were shown in Figure 5C, which indicated that DehpH_H154A, DehpH_S228A, DehpH_D325A, and DehpH_H354A retained 98.65%, 24.31%, 26.92%, and 9.13% enzyme activity, respectively. These results suggested that Ser228, Asp324, and His354 are essential for the catalysis and should be the catalytic triad residues.

## 3. Discussion

It is well known that the isolation of xenobiotic-degrading microbes will provide important sources for the bioremediation of contaminated environments, while the investigation of underlying mechanisms might promote the understanding of the fate of xenobiotics in nature and the development of environmentally friendly biotechnological strategies for bioremediation. The genus *Mycolicibacterium*, previously diverged from the genus *Mycobacterium* by Gupta et al., belongs to the family *Mycobacteriaceae* [19]. Although several species of the genus *Mycobacterium* have been identified as human pathogens, the members of the genus *Mycolicibacterium* are mainly composed of environmental species and primarily non-pathogenic. Additionally, the genus *Mycolicibacterium* is also known as a decomposer that could degrade various xenobiotics, and three isolates of the genus *Mycolicibacterium* capable of degrading PAEs have been reported, including *Mycolicibacterium* sp. MBM [20], *Mycolicibacterium phocaicum* RL-HY01 [18], and *Mycolicibacterium parafortuitum* J101 [21]. As to the known PAE-degrading isolates, most of these isolates are gram-positive bacterial strains (~72.2%) and mainly belong to the phyla of *Actinomycetota*, *Bacillota*, and *Pseudomonadota* (Table S2). Different initial concentrations of substrate were employed for isolation and identification of PAE-degrading microbes, while the present study selected a relatively low concentration of DEHP (50 mg/L) for the functional confirmation of strain RL-HY01. Although the characteristics and metabolic mechanisms of these strains have been preliminarily investigated, all these studies failed to confirm the initial enzyme(s) involved in the metabolic process. Thus, identification and characterization of the enzymes involved in the hydrolysis of the first ester bond would be important and significant for deciphering the molecular mechanisms of PAE degradation in the genus *Mycolicibacterium*.

With the rapid development of sequencing technology, it becomes easier for the scientists to obtain the genetic information of specific microbes, and the interpretation of genomic data would facilitate the understanding about the molecular mechanisms of specific metabolic processes. The primary metabolic pathways of PAEs were divided into two major steps: (a) transformation of PAEs into phthalic acid and (b) the utilization of phthalic acid for cell growth [2]. As the utilization of phthalic acid has been extensively studied, the transformation of PAEs into phthalic acid attracts the researchers’ interests while it is mainly mediated by ester bond hydrolases. Various strategies can be employed for the identification of novel enzymes with specific functions, including the construction of the genomic library, sequence alignment with known functional enzymes, transcriptome analysis, etc. [22,23,24,25]. Whole genome analysis and sequence alignment with known ester bond hydrolases involved in the degradation of PAEs were carried out to identify potential novel PAE hydrolase.

According to the annotation of the genome sequence, approximately 270 hydrolases and 51 esterases were found in the genome of strain RL-HY01. It would be a time- and labor-consuming process to identify the function of these enzymes directly (viz., gene expression and functional confirmation). Therefore, more efficient methods are needed for the fast and preliminary screening of candidate genes. The alignment with known PAE ester bond hydrolases might provide a rapid screening of potential enzymes. The expression level determination of target genes under specific conditions would provide key clues for the discovery of novel functional enzymes. In the present study, seven genes were selected for the expression level determination according to their sequence similarity with known PAEs ester bond hydrolases, and the results indicated that two of these showed relatively high expression levels during the degradation of DEHP. Consequently, these two genes were selected as candidate genes for the following functional confirmation.

The gene cloning and heterologous expression of these two candidate genes further confirmed that pET-RS10760 could efficiently degrade DEHP and was denominated as DehpH. DehpH was identified as a family IV esterase, which is also known as the hormone-sensitive lipase (HSL) family [26]. As shown in Figure 3B, except for XtjR8 (a newly identified esterase capable of hydrolyzing both ester bonds of PAEs) [27], the family IV esterase could only act on the first ester bond of PAEs. The alignment of amino acid sequences demonstrated that typical conserved motifs were identified, including HGGG, YXLAPE, and GXSAGG. Among these motifs, GXSAGG is a well-known pentapeptide motif (GX_1_SX_2_GG) involved in PAE-degrading esterases, and the serine residue in this motif is always recognized as one amino acid residue of the catalytic triad [2]. Additionally, some other conserved motifs or amino acid residues might play an important role in the catalysis process. Nevertheless, the other two amino acid residues of the catalytic triad are still unknown.

DehpH exhibited exceptional catalytic activity across broad temperature (20–50 °C) and pH (5–9) ranges. Similar to the other members of the family IV esterase, DehpH showed preference to the alkaline conditions, and the optimal pH was 8.0 [27,28,29]. The optimal temperature of the members of family IV esterase ranged from 10 °C [30] to 70 °C [28]. The maximum activity of DehpH was determined at 30 °C, and in accord with most esterases of family IV, DehpH is a mesophilic PAEs ester bond hydrolase [27,29,31,32]. Mesophilic enzymes typically exhibit reduced thermal stability compared to thermophilic enzymes at elevated temperatures [23]; the enzyme activity of DehpH decreased dramatically under 50 °C and 60 °C for 5 h. Different metal ions might affect the enzyme activity in different ways, as they might serve as activators or cofactors for enzymes, which could enhance the catalysis process [33,34], or they might also exhibit toxic effects on enzymes [35]. Enzyme tolerance to surfactants and chemicals remains a critical factor in enzymatic biodegradation, particularly for degrading hydrophobic compounds and treating industrial wastewater [36]. Although DehpH can act on short and long side-chain PAEs, DehpH failed to hydrolyze the ester bonds in cyclic side-chain PAE (DCHP), which is inconsist with the previous work [18]. The steric hindrance effects of the catalytic center might be the major reason for the failure of hydrolysis of DCHP [2]. Additionally, the *Km* and *kcat* of DehpH also demonstrated the high efficiency for the degradation of DEHP.

Although some esterases capable of hydrolyzing the ester bonds of PAEs have been identified, most of these studies primarily characterized the enzymes, and only a few illustrated the underlying catalytic mechanisms. Structural modeling and molecular docking are extensively used to predict the interaction between ligand and protein, which would be helpful to elucidate the catalytic mechanisms of specific enzymes. In the present study, the structural modeling and molecular docking predicted the potential amino acid residues of the catalytic triad, and the site-directed mutagenesis further confirmed the catalytic triad. Most of the known reports about PAE ester bond hydrolase preliminarily focused on the identification and characterization, while limited studies have deciphered the underlying catalytic mechanism. Except for the widely reported serine in the motif GXSAGG, Asp324 and His354 were included in the catalytic triad, which was found to be conserved in the esterases of family IV. A similar catalytic triad was observed in EstSP1 [29], XtjR8 [27], and DphB [30], which indicated that Ser228, Asp324, and His354 are functionally indispensable residues forming the catalytic triad.

## 4. Materials and Methods

### 4.1. Chemicals

DEHP (≥98%), DEP (≥99%), and DMP (≥99.5%) were obtained from J&K Scientific (Beijing, China). We prepared a PAE stock solution (20,000 mg/L) in methanol. EDTA, SDS, PMSF, Tween-80, and Triton X-100 were acquired from Sangon Biotech (Shanghai, China); HPLC-grade solvents from Sigma-Aldrich; enzymes/kits from Sangon Biotech (Shanghai, China); and analytical-grade reagents from Sinopharm Chemical Reagent (Shanghai, China).

### 4.2. Stains, Plasmids, and Media

Strain RL-HY01 (*Mycolicibacterium phocaicum*) was obtained from intertidal mangrove sediments in Zhanjiang Bay and has been deposited in the Guangdong Microbial Culture Collection Center (GDMCC) with accession number 61246. *E*. *coli* DH5α competent cells (Takara, Beijing, China) and pMD 19-T (Takara, Beijing, China) were used for subcloning. The recombinant protein was expressed with *E. coli* BL21(D3) (Takara, Beijing, China) and vector pET28a(+) (Novagen, Darmstadt, Germany). pEASY^®^ Pro Seamless Cloning and Assembly Kit (TransGen Biotech, Beijing, China) was applied for the site-directed mutagenesis of the target gene. The primers used in this study are shown in Table 1. Luria-Bertani medium (LB): 10 g/L of peptone, 5 g/L of yeast extracts, and 10 g/L of NaCl, pH 7.0. Mineral salt medium (MSM): 20 g/L of NaCl, 4.8 g/L of NaH_2_PO_4_·12H_2_O, 3.6 g/L of K_2_HPO_4_, 4.5 g/L of KH_2_PO_4_, 1.2 g/L of (NH_4_)_2_SO_4_, 0.1 g/L of MgSO_4_·7H_2_O, 0.05 g/L of FeCl_2_, and 0.03 g/L of CaCl_2_, pH 7.0. The solid medium was obtained by adding 15 g agar per liter.

### 4.3. Degradation Test

A single colony of strain RL-HY01 on an LB plate was inoculated into fresh LB liquid medium, and the culture was incubated under constant shaking (180 rpm and 30 °C). After 24 h’ incubation, 1 mL of the culture was harvested by centrifugation (6000× *g*, 5 min), and the harvested cells were washed by PBS (pH 7.8, 100 mM). The cells were centrifuged and washed three times. Finally, the absorbance of cell suspension at 600 nm (OD_600_) was adjusted to 0.8, and the cell suspension was served as inoculants. The degradation test was conducted in Erlenmeyer flasks (50 mL). Briefly, (i) 1 mL of the inoculants was inoculated into 9 mL of fresh MSM liquid medium; (ii) 10 mL of fresh MSM liquid medium without inoculation was set as an abiotic control; (iii) DEHP was added into each flask to obtain a final concentration of 50 mg/L; (iv) all treatments were conducted in three replicates; (v) all samples underwent continuous shaking incubation (180 rpm, 30 °C); and (vi) DEHP residues were quantified via gas chromatography at 12 h intervals using established methods [18].

### 4.4. Genomic DNA Extraction, Sequencing, and Annotation

A single RL-HY01 colony from an LB plate was used to inoculate fresh LB broth and cultured at 30 °C with 180 rpm shaking for 24 h. Cells were harvested via centrifugation (6000× *g*, 5 min), washed with PBS buffer (0.1 M, pH 7.8), and subjected to genomic DNA extraction using the TaKaRa MiniBEST kit (Takara, Beijing, China). DNA purity, integrity, and concentration were assessed, respectively, by Nanodrop 2000, 0.35% agarose gel electrophoresis, and Qubit. MinION sequencing (Oxford Nanopore, Oxford, UK) was performed by Biomarker Technologies (Beijing, China), followed by de novo assembly with Canu v1.5 [37].

The assembled genome was processed through PGAP for annotation (PGAP, https://www.ncbi.nlm.nih.gov/refseq/annotation_prok/, accessed on 18 October 2021) and the Rapid Annotation using Subsystem Technology server (RAST, https://rast.nmpdr.org/, accessed on 18 October 2021) for automatic gene prediction and in-depth annotation [38,39]. Functional annotation of predicted CDSs was performed against multiple databases: the Non-Redundant Protein database (NR), the Gene Ontology database (GO), the Kyoto Encyclopedia of Genes and Genomes database (KEGG), eggNOG, Pfam, and SwissProt. Concurrently, genomic islands (GIs) were identified using IslandViewer 4 [40]. The circular representation of the genome was generated by Proksee [41]. BLAST-based whole genome screening of potential enzymes involved in the hydrolysis of ester bonds in PAEs was conducted with BLASTP (version 2.2.26) with an e-value cutoff of 10^−3^ and a sequence similarity above 40%.

### 4.5. RNA Isolation and RT-qPCR Analyses

According to the genome annotation and alignments with known enzymes involved in the hydrolysis of ester bonds in PAEs, seven candidate genes were selected for the RT-qPCR analyses. The detailed information of these seven candidate genes was shown in Table S1, and the primers for the RT-qPCR analyses were listed in Table 1. The primer efficiency was validated through standard curve analysis, and product specificity was confirmed by melting curve analysis. Two treatments were set up for the total RNA isolation, including (i) MSM with 50 mg/L of DEHP and (ii) MSM with 5 g/L of glucose. The inoculants of strain RL-HY01 were prepared as described above and inoculated into these two treatments. All cultures were incubated under constant shaking (180 rpm, 30 °C), and all treatments were performed in triplicate. After 24 h’ incubation, the cells of strain RL-HY01 were harvested for the extraction of total RNA using a TRIzol total RNA extraction kit (Transgene Biotech, Beijing, China). During the isolation of total RNA, DNA was digested using RNase-free DNase I (37 °C, 1 h). The total RNA was quantified with a NanoDrop 2000 spectrophotometer at 260 nm, while the purity was estimated from the 260/280 absorbance ratio. The integrity of the extracted RNA was analyzed through agarose (2.0%, *w*/*v*) gel electrophoresis. The real-time quantitative PCR (RT-qPCR) was performed in 96-well RT-qPCR plates using a CFX Connect™ Real-Time PCR System (Bio-Rad, Hercules, CA, USA). The first-strand cDNA was prepared using a cDNA Synthesis SuperMix Kit, while the qPCR was accomplished with ChamQ Blue Universal SYBR qPCR Master Mix (Vazyme, Nanjing, China) according to the manufacturer’s instructions. The 16S rRNA gene was kept as an endogenous control for each set of reactions, and the primers are shown in Table 1. The PCR program was set as follows: 94 °C for 30 s (pre-denaturation), followed by 45 cycles of 94 °C for 5 s (denaturation) and 60 °C for 30 s (primer hybridization and polymerization). Finally, the relative gene expression level of specific genes was calculated using the comparative threshold amplification cycle via the 2^−ΔΔCt^ method [20].

### 4.6. Cloning and Heterologous Expression of Candidate Genes

Through gene annotation and RT-qPCR analyses, the genes encoding enzymes potentially involved in the hydrolysis of ester bonds were selected for cloning and expression. Primers for gene cloning were listed in Table 1. The genomic DNA of strain RL-HY01 was prepared as described above. Taq PCR Master Mix (Sangon Biotech, Shanghai, China) was employed for the amplification of target genes. The PCR reaction solution was composed of Taq PCR Master Mix (25 μL), template DNA (1 μL), forward primer (2 μL), reverse primer (2 μL), and ddH_2_O (20 μL). The cycling program was set as follows: (a) 94 °C for 4 min, (b) 35 cycles of 94 °C for 30 s, 55 °C for 30 s, and 72 °C for 80 s, (c) 72 °C for 10 min, and (d) 4 °C for 2 h. The PCR products were analyzed by 0.8% (*w*/*v*) agarose gel electrophoresis, purified using the TaKaRa MiniBEST Agarose Gel DNA Extraction Kit Ver.4.0, inserted into the pMD19-T vector, and finally sequenced by Shanghai Sangon Biotech (Shanghai, China).

For the construction of expression plasmids, the plasmids of positive transformants and pET28a(+) were digested with specific restriction enzymes, while the restriction sites in the primers are shown in Table 1. Candidate gene fragments were subsequently recovered and subcloned into the pET28a(+) vector containing an N-terminal Trx-His6 tag, generating pET-(gene) constructs. These plasmids were transformed into *E. coli* BL21(DE3) competent cells, with positive transformants verified by sequencing (Sangon Biotech, Shanghai, China).

For the heterologous expression of candidate genes, the colonies of positive transformants were inoculated into liquid LB broth and incubated under constant shaking (37 °C, 160 rpm) until OD_600_ reached 0.8. Cultures of *E*. *coli* BL21 (DE3) containing the plasmid of pET28a(+) were set as the control treatment (CK). Subsequently, DEHP was added into the culture with a final concentration of 100 mg/L, and the cells were induced by isopropyl-D-thiogalactopyranoside (IPTG, 1 mM) under 16 °C for 8 h. Finally, the residual concentration of DEHP in cultures was determined by GC. All treatments were conducted in triplicate. The enzyme capable of hydrolyzing the ester bond of DEHP was denominated as di-(2-ethylhexyl) phthalate hydrolase (DehpH).

### 4.7. Sequence Analysis and Purification of Recombinant DehpH

The phylogenetic analysis of DehpH and known hydrolases of PAE ester bonds was performed with MEGA 11.0 by the neighbor-joining method (Bootstrap value of 1000) [42]. The detailed information of these hydrolases was shown in Table S2. Further, multiple sequence alignment between the target enzyme and its related family members was conducted with ESPript 3.0 (http://espript.ibcp.fr, accessed on 20 March 2025) to analyze the conserved amino acid residues and motifs [43].

To express *dehpH*, we transferred a single recombinant colony (pET-dehpH) into LB broth and cultured it at 37 °C with 160 rpm shaking until OD_600_ reached 0.8. Cells were subsequently induced with 1 mM IPTG at 16 °C for 8 h. Four control treatments were set as below: (a) *E*. *coli* BL21(DE3) without inducing, (b) *E*. *coli* BL21(DE3)-pET28a(+) without inducing, (c) *E*. *coli* BL21(DE3)-pET28a(+) induced by IPTG, and (d) *E*. *coli* BL21(DE3)-pET-dehpH without inducing. The cells were harvested by centrifuging (6000× *g*, 5 min) and washed twice, and then resuspended in Tractor Buffer provided by Takara (Beijing, China). The cells were lysed by ultrasonication in an ice and water mixture (repeating cycles of 5 s on and 10 s off, sustained for 20 min). Subsequently, the lysate was then centrifuged at 10,000× *g* under 4 °C for 10 min, and the supernatant was used for purification. The protein of DehpH was purified using His60 Ni Magnetic Beads (Takara, Beijing, China) according to the manufacture’s guide. Finally, the protein extracts were analyzed by sodium dodecyl sulfate-polyacrylamide gel electrophoresis (SDS-PAGE).

### 4.8. Biochemical Characterization of DehpH

The activity of recombinant DehpH was analyzed by monitoring the hydrolysis of DEHP in a 2 mL reaction mixture consisting of 200 μM DEHP and 20 μg purified enzyme. DEHP in the mixture was extracted and quantified with a gas chromatograph as previously described [18]. A DehpH unit represents the amount of enzyme catalyzing the consumption of 1 μmol DEHP per minute. The optimal pH for enzyme activities was determined in different buffers: pH 3.0–7.0, 50 mM Na_2_HPO_4_-citric acid buffer; pH 7.0–9.0, 50 mM Tris-HCl buffer; and pH 9.0–10.0, 50 mM Glycine-NaOH buffer. DehpH’s pH stability was assessed via residual activity measurements following 1 h pre-incubation of purified enzyme at different pH values (4 °C) (without supplementation of DEHP). Similarly, the optimal temperature was determined under a range of temperatures (10 to 80 °C, with an interval of 10 °C) in 50 mM Tris-HCl buffer (pH 8.0) for 10 min. The thermostability of DehpH was evaluated by measuring the residual activity after preincubation of the enzyme under different temperatures (20 °C to 60 °C) for 1 to 5 h (without supplementation of DEHP). The effects of metal ions (1 mM) on the activity of DehpH were investigated with Ca^2+^, Co^2+^, Cu^2+^, Fe^2+^, Mg^2+^, Mn^2+^, and Zn^2+^. Assays were conducted in 50 mM Tris-HCl buffer (pH 8.0), and the reaction mixture without metal ion was set as the control treatment (CK). All the treatments were incubated under the optimized conditions for 10 min, and the enzyme activity was determined thereafter. The effects of typical surfactants (including EDTA, SDS, PMSF, Tween-80, and Triton X-100) and chemicals (including methanol, ethanol, isopropanol, acetone, acetonitrile, ethyl acetate, and *n*-hexane) on the enzyme activity were analyzed by adding the surfactant or chemical into the reaction mixture with a ratio of 1.0% (*v*/*v*). The reaction mixture without surfactant and these chemicals was set as the control treatment (CK). All treatments were incubated under the optimized conditions for 10 min, and the enzyme activity was determined thereafter. Substrate specificity of DehpH was investigated with typical short side-chain PAEs (di-methyl phthalate, DMP; and di-ethyl phthalate, DEP), long side-chain PAEs (DEHP and di-n-octyl phthalate, DnOP), and cyclic side-chain PAEs (DCHP) as substrates. The reaction mixture contained 1 mL of 50 mM Tris-HCl (pH 8.0), 200 μM PAE, and 20 μg purified enzyme. Mixtures without enzyme served as controls. All treatments underwent incubation at 30 °C for 10 min before measuring residual PAE concentration.

Kinetics parameters were determined following Michaelis–Menten kinetics using DEHP as a substrate [44]. A series of reaction mixtures (1 mL, 50 mM Tris-HCl, pH 8.0) with different concentrations of DEHP were prepared, including 50 μM, 125 μM, 500 μM, 1000 μM, 1500 μM, and 2000 μM. Subsequently, 20 μg of purified enzyme was added, and all reaction mixtures were incubated at 30 °C for 10 min. The residual concentration of PAE was determined, and the kinetic parameters were calculated using the function of non-linear fitting of software Origin 2025 (internally installed Michaelis–Menten function).

### 4.9. Structural Modeling and Molecular Docking of DehpH

Three-dimensional models of DehpH were predicted by SWISS-MODEL (https://swissmodel.expasy.org/, accessed on 7 June 2024) and I-TASSER (https://zhanggroup.org//I-TASSER/, accessed on 7 June 2024). The obtained models were checked by SAVEs v6.0 (https://saves.mbi.ucla.edu/, accessed on 7 June 2024), and the model with the best validation scores was selected for further study. DEHP has a long and branched side-chain, which makes the variability of the side-chain structure exceed the limitations and lead to the failure of docking. Considering the flexibility of the side-chain and the degrading capability of DMP, DMP was selected as the ligand, and the ligand file was downloaded from PubChem Compound (https://pubchem.ncbi.nlm.nih.gov/compound/8554, accessed on 7 June 2024). Molecular docking of receptor DehpH and ligand DMP was conducted with Autodock 4.2.6 [45]. The conformation with the lowest free energy was selected to analyze the protein-ligand interactions. The results were visualized by Pymol (version 1.5.0.3) and the amino acid residues potentially involved in the catalysis were identified.

### 4.10. Site-Directed Mutagenesis of the Active-Site Residues

The site-directed mutagenesis of potential catalytic residues was performed with a pEASY^®^-Basic Seamless Cloning and Assembly Kit (TransGen Biotech Co., Ltd., Beijing, China). The primers used for mutagenesis were listed in Table 1. The mutant plasmids were transferred into *E*. *coli* BL21 (DE3) and confirmed by DNA sequencing. The transformants were grown at 37 °C in LB agar plates (100 mg/L DEHP) and further analyzed by enzymatic activity assay as described above.

### 4.11. Accession Number

Strain RL-HY01 has been deposited in the Guangdong Microbial Culture Collection Center (GDMCC) with accession number 61246. The 16S rRNA gene and complete genome sequence of strain RL-HY01 are available from GenBank with accession numbers MK787328 and CP084713, respectively.

## 5. Conclusions

In summary, a novel di-(2-ethylhexyl) phthalate hydrolase gene (dehpH) was identified from a previously isolated PAEs-degrading marine bacterial strain, *Mycolicibacterium phocaicum* RL-HY01. Gene *dehpH* was cloned, expressed, and characterized, and the results demonstrated the application potential of DehpH. Further, sequence alignment, structural modeling, and molecular docking were conducted to have an insight into catalytic process and mechanisms of DehpH-mediated PAE degradation. Furthermore, mutants of DehpH with restrained activity indicated that Ser228, Asp324, and His354 residues were essential in the process of catalysis. The identification and characterization of dehpH advance our understanding of the catalytic mechanisms of PAE biodegradation and the fate of PAEs in marine ecosystems. In addition, this study also provides gene resources for bioremediation of the environmental pollution caused by PAEs.

## Figures and Tables

**Table 1:** Primers used in this study.

Target Fragments	Forward Primer (5′–3′)	Reverse Primer (5′–3′)	Objectives
RS10760	CCGATGCTGGTCTTCTTCCA	CCAGAGATAGGTGGCGTAGC	RT-qPCR
RS11055	GACCAGTACGTCCCCAACAC	GGATGGCTCCGTAGTACAGC	
RS12955	GACGGACTCACGCTGAAAGA	ATGGTGTCGGTGACGAGTTC	
RS13175	CCATCACCGTCGAGGATCTG	GTCGACTGGCTGTAGGTCAG	
RS13285	GGAAGCGGTTCCCTTCCAAT	TGGAAGAACAGCACGACCG	
RS13880	TATCGACAGCGGAACCATCG	ACGCTCAGCATCGTGTACTT	
RS21765	CAGAAGCTGGTCGGGATCG	ATGTGGGCGGAACGTAGTG	
16S rRNA gene	AGAGTTTGATCCTGGCTCAG	GGTTACCTTGTTACGACTT	
RS10760	GCGC GAATTC ACCATGAGCATGCCAGGCGA	GCGC AAGCTT GCGGGTGAGGTGCGCCCGG	Gene expression
RS21765	GCGC GGATCC ACTGCGAGTCTGCCAGCTGACG	GCGC GAATTC GACGCCGCGCAGACGGGTGCGCAGC	
H154up	GCTGATATCG GATCC ACCATGAGCATGCCAGG	GACGAATCCGCCGCC GGC GAAGAAGACCAGCAT	Site-directed mutagenesis (H154A)
H154down	ATGCTGGTCTTCTTC GCC GGCGGCGGATTCGTC	GAGTGCGGCCGCAAGCTT GCGGGTGAGGTGCGCCC	
S228up	GCTGATATCG GATCC ACCATGAGCATGCCAGG	GCCGCCGGC GGC GTCGCCGCCGACTG	Site-directed mutagenesis (S228A)
S228down	GCGACGCC GCCGGCGGCAACCTGGCG	GAGTGCGGCCGC AAGCTT GCGGGTGAGGTGCGCCC	
D324up	GCTGATATCGGATCC ACCATGAGCATGCCAGG	GTCGCGCAGCGG GGC GAACCCACCGGT	Site-directed mutagenesis (D324A)
D324down	ACCGGTGGGTTC GCC CCGCTGCGCGAC	GAGTGCGGCCGCAAGCTT GCGGGTGAGGTGCGCCC	
H354up	GCTGATATCGGATCC ACCATGAGCATGCCAGG	ATTCGCGAAGGC GGC GATCAGGGACCC	Site-directed mutagenesis (H354A)
H354down	GGGTCCCTGATC GCC GCCTTCGCGAAT	GAGTGCGGCCGCAAGCTT GCGGGTGAGGTGCGCCC	

The restriction sites in the primers are underlined; the mutated codons are marked with a gray background.

## Data Availability

The data supporting this research are included in the article and are available upon request to interested parties.

## Supplementary Materials

The following supporting information can be downloaded at https://www.mdpi.com/article/10.3390/ijms26178141/s1. References [27,46,47,48,49,50,51,52,53,54,55,56,57] are cited in the supplementary materials.
