# Supplementary material for: Identification and Characterization of a Novel Di-(2-ethylhexyl) Phthalate Hydrolase from a Marine Bacterial Strain Mycolicibacterium phocaicum RL-HY01
Source: Int J Mol Sci. 2025 Aug 22;26(17):8141. doi: 10.3390/ijms26178141 (PMC12428281; doi:10.3390/ijms26178141)
Supplement: Supplementary file 1 [file ijms-26-08141-s001.zip › ijms-3793518-supplementary.pdf]

Identification and characterization of a novel di-(2-ethylhexyl) phthalate hydrolase from a marine bacterial strain *Mycolicibacterium phocaicum* RL-HY01

Lei Ren[a], Caiyu Kuang[a], Hongle Wang[a], John L. Zhou[b], Min Shi[a], Danting Xu[a], Hanqiao Hu[a], Yanyan Wang[a, *]

a College of Coastal Agricultural Sciences, Guangdong Ocean University, Zhanjiang 524088, China

b Faculty of Science and Engineering, University of Nottingham Ningbo China, Ningbo 315100, China

* Corresponding authors:

Yanyan Wang, Guangdong Ocean University, e-mail: yanyanwang@gdou.edu.cn

Table S1 Candidate enzymes potentially involved in the hydrolysis of ester bonds in PAEs

ID	Accession number	Annotation	Length (aa)
RS12955	LHJ73_RS12955	alpha/beta hydrolase fold domain-containing protein	860
RS10760	LHJ73_RS10760	alpha/beta hydrolase	388
RS21765	LHJ73_RS21765	alpha/beta hydrolase	371
RS11055	LHJ73_RS11055	alpha/beta hydrolase	309
RS13285	LHJ73_RS13285	alpha/beta hydrolase	311
RS13880	LHJ73_RS13880	carboxylesterase/lipase family protein	510
RS13175	LHJ73_RS13175	serine hydrolase domain-containing protein	409

Tabel S2 The detailed information of PAEs ester bond hydrolases used in this study

Enzymes	Accession No.	Sources	References
PAEs hydrolase	AAK16532	*Arthrobacter keyseri* 12B	[46]
Triacylglycerol lipase	AEW03609	*Sulfobacillus acidophilus* DSM10332	[28]
Dialkyl PEs hydrolase	AFK31309	*Acinetobacter* sp. M673	[31]
Dialkyl PEs hydrolase	AGY55959	Metagenome	[30]
Dialkyl PEs hydrolase	AGY55960	Metagenome	[30]
Esterase (EstB)	AJO67803	*Sphingobium* sp. SM42	[47]
Esterase (EstG)	AJO67804	*Sphingobium* sp. SM42	[47]
Lipase (EstSP1)	WP010186968	*Sphingomonas* sp. PAMC 26605	[29]
MEHP hydrolase	BAE78500	*Gordonia* sp. P8219	[48]
Phthalate ester hydrolase	ABG99214	*Rhodococcus jostii* RHA1	[49]
Phthalate ester hydrolase	ABH00399	*Rhodococcus jostii* RHA1	[49]
MEHP hydrolase	AMJ52171	*Gordonia* sp. YC-RL2	[50]
MEHP hydrolase	BAU22081	*Rhodococcus* sp. EG-5	[51]
Carboxylesterase	AIZ00845	*Bacillus* sp. K91	[52]
Dhs82	APM86632	Metagenome	[32]
XtjR8	QFQ13832	Metagenome	[27]
GoEst15	AYW76487	*Gordonia sp. strain* 5F	[53]
p-nitrobenzyl esterase	WWO58214	*Bacillus velezensis* NP05	[54]
HylD1	F8A10_04470	*Paracoccus kondratievae* BJQ0001	[55]
BaCEs04	QEO33334	*Bacillus velezensis* SYBC H47	[56]
EstM2	AJG42113	Metagenome	[57]
DEHP hydrolase	LHJ73_RS10760	*Mycolicibacterium phocaicum* RL-HY01	This study
MEHP hydrolase	LHJ73_RS21370	*Mycolicibacterium phocaicum* RL-HY01	This study

Table S3 General features of *Mycolicibacterium phocaicum* RL-HY01

Items	Description
**General feature**	
Classification	Domain Bacteria
	Phylum Actinobacteria
	Class Actinobacteria
	Order Corynebacteriales
	Family Mycobacteriaceae
	Genus Mycolicibacterium
Gram stain	Positive
Cell shape	Rod
Pigmentation	Cream white
pH tolerance	5.0-9.0
Salinity	0.0%-9.0% (w/v)
Temperature tolerance	20°C-40°C
**MIGS data**	
BioProject	PRJNA769661
BioSample	SAMN22157750
Geographic location	Zhanjiang Bay, China
Latitude and longitude	21°215′418″N, 110°427′797″E
Environment (biome)	marine biome ENVO:00000447
Environment (feature)	marine sediment ENVO:03000033
Environment (material)	subtidal marine sediment ENVO:03000034
Environmental package	Sediment
Collection time	May, 2018
**Genome characteristics**	
Sequencing Technology	Oxford Nanopore MinION
Assembly method	Canu v. 1.5
Genome coverage	255×
Size(bp)	6,064,759
DNA G+C content (mol %)	66.93%
CDSs	5,874
tRNAs	57
16s-23s-5s rRNAs	2-2-2
Finishing quality	Complete genome
Submitted to NCBI	October, 2021

Table S4 The annotation of predicted coding sequences (CDSs) against different databases

Database	Number	100<=length<300	length>=300
eggNOG_Annotation	4,591	2,106	2,327
GO_Annotation	3,938	1,782	2,009
kegg_Annotation	2,002	755	1,179
nr_Annotation	5,762	2,789	2,579
Pfam_Annotation	4,722	2,191	2,365
Swissprot_Annotation	3,114	1,232	1,789
TrEMBL_Annotation	3,114	1,232	1,789
All_Annotated	5,764	2,789	2,579
